# Supplementary material for: Candida albicans evades NK cell elimination via binding of Agglutinin-Like Sequence proteins to the checkpoint receptor TIGIT
Source: Nat Commun. 2022 May 5;13:2463. doi: 10.1038/s41467-022-30087-z (PMC9072312; doi:10.1038/s41467-022-30087-z)
Supplement: Supplementary file 1 — Supplementary Information [file 41467_2022_30087_MOESM1_ESM.pdf]

# *Supplementary Information*

## ***Candida albicans*** **evades NK cell elimination via binding of Agglutinin-Like Sequence proteins to the checkpoint receptor TIGIT**

Yoav Charpak-Amikam[1], Tom Lapidus[1], Batya Isaacson[1], Alexandra Duev-Cohen[1], Tal Levinson[2], Adi Elbaz[3], Francesca Levi-Schaffer[4], Nir Osherov[5], Gilad Bachrach[6], Lois L Hoyer[7], Maya Korem[8], Ronen Ben-Ami[2], and Ofer Mandelboim[1,*]

* Corresponding author: Ofer Mandelboim, Tel: 972-2-6757515/6, Fax: 972-2-6424653. Email: oferm@ekmd.huji.ac.il

# Supplementary figure 1

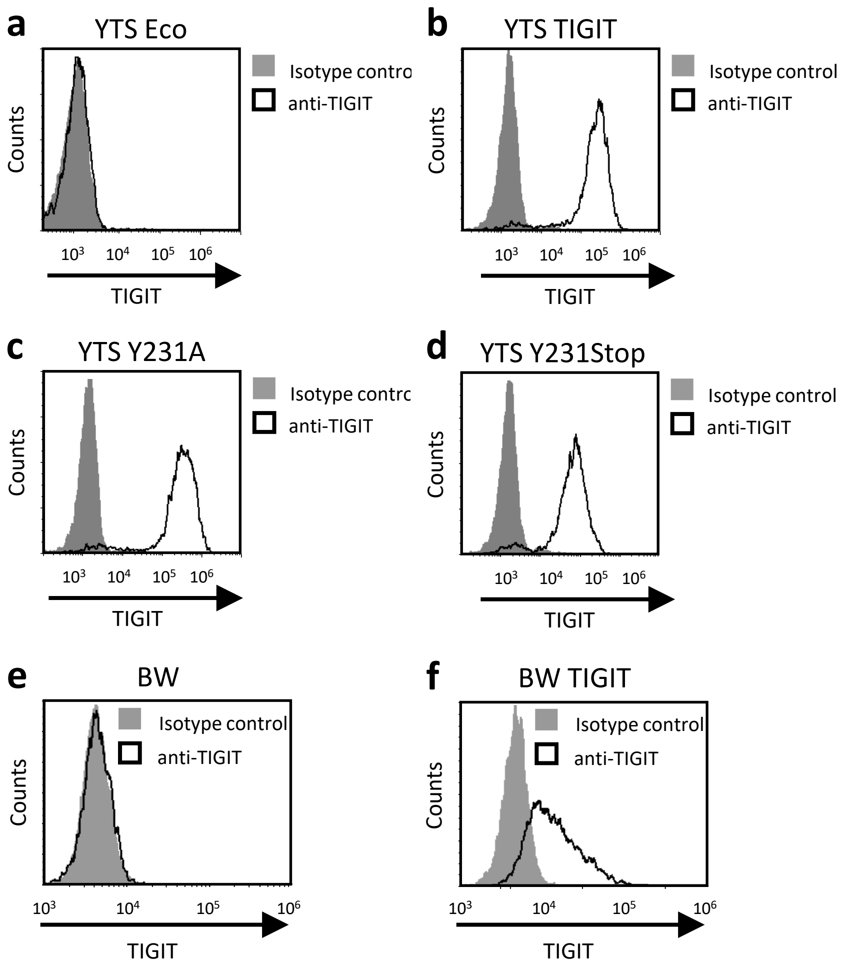


**Supplementary figure 1, validation of transgene expression on selected cell lines. Related to figures 2 and 3**

**A-F.** Flow cytometry stainings using an anti-TIGIT antibody (Black empty histogram) or an isotype control antibody (filled grey histogram). One representative experiment out of 3 is presented. The cell lines stained were YTS Eco (A), YTS TIGIT (B), YTS TIGIT Y231A (C), YTS TIGIT T231Stop (D), BW (E) or BW-TIGIT (F).

# Supplementary figure 2

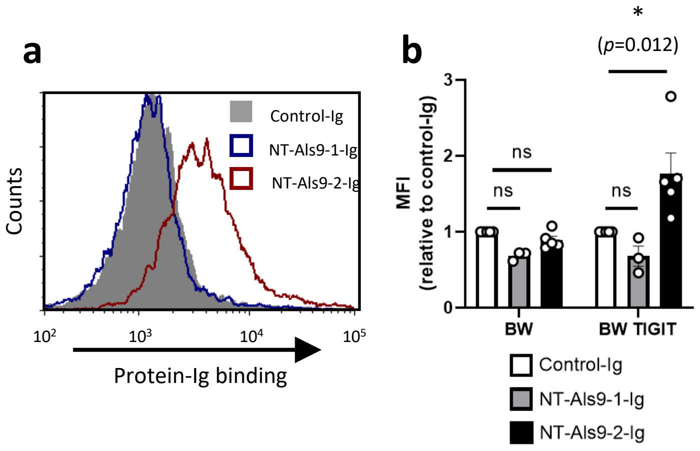


**Supplementary figure 2, Als9 alleles bind TIGIT differentially.**

**Related to figure 3**

**A.** Flow cytometry staining of BW and BW TIGIT cells using NT-Als9-1-Ig (blue empty histogram), NT-Als9-2-Ig (red empty histogram) or a negative control protein (filled grey histogram). One representative experiment out of 3-5 is presented. **B.** Quantification of the results presented in A. n = 3-5 independent experiment. Data are presented as mean values +/- SEM. Significance was tested using a two-tailed Student's T test. ns = not-significant, * = $p < 0.05$.

# Supplementary figure 3

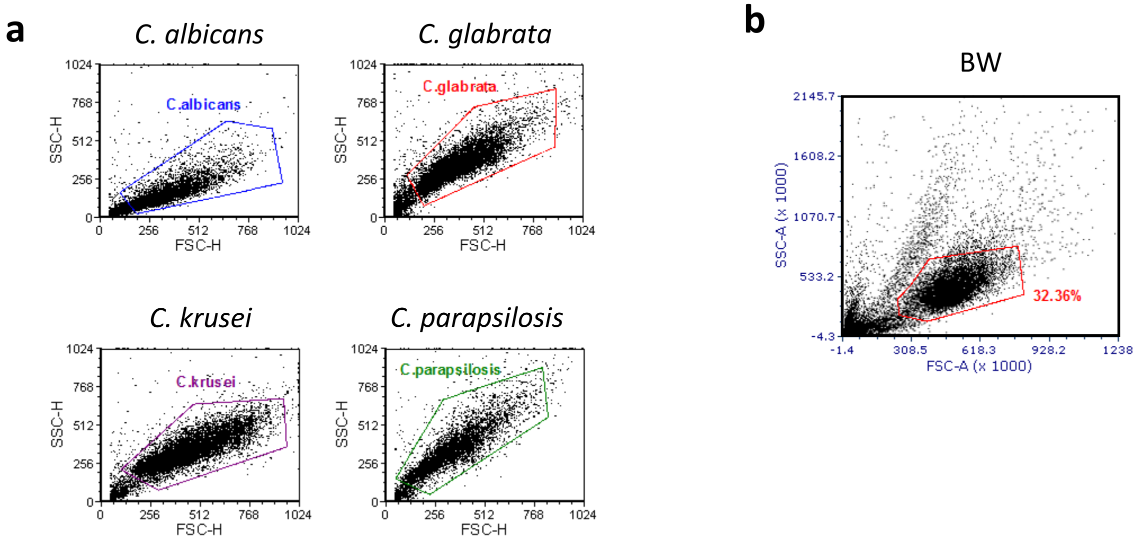


**Supplementary figure 3, Gating strategy. Related to figures 1-4**

**A**. A scatter plot demonstrating the gating strategy used to analyze Candida species cell stainings in flow cytometry experiments. One representative plot is presented. **B.** A scatter plot demonstrating the gating strategy used to analyze BW cell stainings in flow cytometry experiments. One representative plot is presented.
