# Supplementary material for: Candida albicans evades NK cell elimination via binding of Agglutinin-Like Sequence proteins to the checkpoint receptor TIGIT
Source: Nat Commun. 2022 May 5;13:2463. doi: 10.1038/s41467-022-30087-z (PMC9072312; doi:10.1038/s41467-022-30087-z)
Supplement: Supplementary file 3 — Description of Additional Supplementary Files [file 41467_2022_30087_MOESM3_ESM.pdf]

Description of Additional Supplementary Files

Supplementary Data 1

Supplementary Data 1 provides the full nucleotide sequences used to clone The Ig-fusion proteins Als6-NT-Ig, Als7-NT-Ig, Als9-1-NT-Ig and Als9-2-NT-Ig, used in figures 3c, 3d and 4 and in supplementary figure 2.

The sequences are color-coded as described next:

Green: CD5 signal peptide

Red: BamHI recognition site

Yellow: EcoRI recognition site

Lowercase black: aaaa linker

Blue: Last nucleotide was replaced with a G for compatibility with the BamHI cut site
