# Supplementary material for: Candida albicans evades NK cell elimination via binding of Agglutinin-Like Sequence proteins to the checkpoint receptor TIGIT
Source: Nat Commun. 2022 May 5;13:2463. doi: 10.1038/s41467-022-30087-z (PMC9072312; doi:10.1038/s41467-022-30087-z)
Supplement: Supplementary file 5 — Reporting Summary [file 41467_2022_30087_MOESM5_ESM.pdf]

nature portfolio

Corresponding author(s): Ofer Mandelboim
Last updated by author(s): Mar 5, 2022

Reporting Summary

Nature Portfolio wishes to improve the reproducibility of the work that we publish. This form provides structure for consistency and transparency in reporting. For further information on Nature Portfolio policies, see our Editorial Policies and the Editorial Policy Checklist.

Statistics

For all statistical analyses, confirm that the following items are present in the figure legend, table legend, main text, or Methods section.

n/a	Confirmed	
☐	☒	The exact sample size (n) for each experimental group/condition, given as a discrete number and unit of measurement
☐	☒	A statement on whether measurements were taken from distinct samples or whether the same sample was measured repeatedly
☐	☒	The statistical test(s) used AND whether they are one- or two-sided<br>*Only common tests should be described solely by name; describe more complex techniques in the Methods section.*
☐	☒	A description of all covariates tested
☐	☒	A description of any assumptions or corrections, such as tests of normality and adjustment for multiple comparisons
☐	☒	A full description of the statistical parameters including central tendency (e.g. means) or other basic estimates (e.g. regression coefficient) AND variation (e.g. standard deviation) or associated estimates of uncertainty (e.g. confidence intervals)
☐	☒	For null hypothesis testing, the test statistic (e.g. F, t, r) with confidence intervals, effect sizes, degrees of freedom and P value noted<br>*Give P values as exact values whenever suitable.*
☐	☒	For Bayesian analysis, information on the choice of priors and Markov chain Monte Carlo settings
☐	☒	For hierarchical and complex designs, identification of the appropriate level for tests and full reporting of outcomes
☒	☐	Estimates of effect sizes (e.g. Cohen's d, Pearson's r), indicating how they were calculated

Our web collection on statistics for biologists contains articles on many of the points above.

Software and code

Policy information about availability of computer code

Data collection	No software was used for data collection
Data analysis	Microsoft® Excel® for Microsoft 365 MSO (Version 2112 Build 16.0.14729.20312) 64-bit and GraphPad Prism (Version 9.3.1 (471)) were used for data analysis

For manuscripts utilizing custom algorithms or software that are central to the research but not yet described in published literature, software must be made available to editors and reviewers. We strongly encourage code deposition in a community repository (e.g. GitHub). See the Nature Portfolio guidelines for submitting code & software for further information.

Data

Policy information about availability of data

All manuscripts must include a data availability statement. This statement should provide the following information, where applicable:

- Accession codes, unique identifiers, or web links for publicly available datasets
- A description of any restrictions on data availability
- For clinical datasets or third party data, please ensure that the statement adheres to our policy

All important data generated and analyzed in the context of the present study are included within the article. Source data are provided with this paper.

Field-specific reporting

Please select the one below that is the best fit for your research. If you are not sure, read the appropriate sections before making your selection.

[x] Life sciences [ ] Behavioural & social sciences [ ] Ecological, evolutionary & environmental sciences

For a reference copy of the document with all sections, see nature.com/documents/nr-reporting-summary-flat.pdf

Life sciences study design

All studies must disclose on these points even when the disclosure is negative.

Sample size	No sample-size calculations were performed. All experiments were planned as to contain sufficient technical and biological repeats in accordance with the experimenters' prior experience, the observed technical and biological variability, and the accepted norms in the field
Data exclusions	Outliers in the data sets were excluded after identification using Grubbs's test with an alpha of 0.05
Replication	All experiments were successfully repeated at least once. The experiment containing Candida isolates generated from clinical samples of patients was not repeated per se, but was performed during several independent dates and contains over 100 independent samples from different patients which constitute their own biological replicates
Randomization	For the animal experiments, mice were randomly allocated to the different experimental groups.
Blinding	Data acquisition and analysis were done in a non-blinded fashion. Blinding was deemed as irrelevant as all of the experimental outcomes were objective and not subjected to observer bias (for examples MFI values generated by flow cytometry or a number of colonies generated from a mouse organ).

Reporting for specific materials, systems and methods

We require information from authors about some types of materials, experimental systems and methods used in many studies. Here, indicate whether each material, system or method listed is relevant to your study. If you are not sure if a list item applies to your research, read the appropriate section before selecting a response.

Materials & experimental systems

n/a	Involved in the study	
[ ]	[x]	Antibodies
[ ]	[x]	Eukaryotic cell lines
[x]	[ ]	Palaeontology and archaeology
[ ]	[x]	Animals and other organisms
[ ]	[x]	Human research participants
[x]	[ ]	Clinical data
[x]	[ ]	Dual use research of concern

Methods

n/a	Involved in the study	
[x]	[ ]	ChIP-seq
[ ]	[x]	Flow cytometry
[x]	[ ]	MRI-based neuroimaging

Antibodies

Antibodies used

Anti-human CD56-PE BioLegend (ENCO Israel) 318306, clone HCD56
Anti-human CD3-FITC BioLegend (ENCO Israel) 300306, clone HIT3a
Anti-human CD4-PE BioLegend (ENCO Israel) 357404, clone A161A1
Anti-human TIGIT-APC BioLegend (ENCO Israel) 372706, clone A15153G
Anti-human CD3 BioLegend (ENCO Israel) 300314, clone HIT3a
Anti-human IFN BioLegend (ENCO Israel) 502402, clone NIB42
Biotinylated Anti-human IFN BioLegend (ENCO Israel) 502504, clone 4S.B3
Anti-mouse IL-2 BioLegend (ENCO Israel) 503702, JES6-1A12
Biotinylated Anti-mouse IL-2 BioLegend (ENCO Israel) 503804, JES6-5H4
InVivoMab anti-mouse TIGIT BioXCell (ENCO Israel) BE0274, clone 1G9
InVivoMab anti-mouse NK1.1 BioXCell (ENCO Israel) BE0036, clone PK136
InVivoMab anti-mouse CD3 BioXCell (ENCO Israel) BE0002, clone 17A2
Biotinylated Anti-human IgG Jackson ImmunoResearch (ENCO Israel) 309-065-082, polyclonal
Anti-human-AlexaFluor 647 Jackson ImmunoResearch (ENCO Israel) 709-606-098, polyclonal
Anti-mouse-AlexaFluor 647 Jackson ImmunoResearch (ENCO Israel) 115-606-062, polyclonal
Anti-human-APC Jackson ImmunoResearch (ENCO Israel) 709-136-098, polyclonal
Mouse IgG1 Isotype Control BioLegend (ENCO Israel) 400102, clone MPOC-21
Mouse IgG1-PE Isotype Control BioLegend (ENCO Israel) 400112, clone MPOC-21

Mouse IgG2a-FITC Isotype Control BioLegend (ENCO Israel) 400208, clone MOPC-173
Rat IgG2b-PE Isotype Control BioLegend (ENCO Israel) 400608, clone RTK4530
Mouse IgG2a-APC Isotype Control BioLegend (ENCO Israel) 400220, clone MOPC-173
Mouse IgG2a Isotype Control BioLegend (ENCO Israel) 400202, clone MOPC-173
Mouse IgG1 Anti-human TIGIT, monoclonal, In-house(Stanietsky et al., 2009, PMID 19815499)

Validation

The IgG1 anti-human TIGIT was developed and prepared in-house and validated in Stanietsky et al., 2009, PMID 19815499.
All primary antibodies other than mouse IgG1 anti-human TIGIT were commercial antibodies with quality control and validation statements provided on their manufacturer's website:
Anti-human CD56-PE BioLegend: https://www.biolegend.com/en-us/products/pe-anti-human-cd56-ncam-antibody-3796
Anti-human CD3-FITC BioLegend: https://www.biolegend.com/en-us/products/fitc-anti-human-cd3-antibody-751
Anti-human CD4-PE BioLegend: https://www.biolegend.com/en-us/products/pe-anti-human-cd4-antibody-8511
Anti-human TIGIT-APC BioLegend: https://www.biolegend.com/en-us/products/apc-anti-human-tigit-vstm3-antibody-13758
Anti-human CD3 BioLegend: https://www.biolegend.com/en-us/products/ultra-leaf-purified-anti-human-cd3-antibody-7741
Anti-human IFN BioLegend: https://www.biolegend.com/en-us/products/purified-anti-human-ifn-gamma-antibody-1354
Biotinylated Anti-human IFN BioLegend: https://www.biolegend.com/en-us/products/biotin-anti-human-ifn-gamma-antibody-1014
Anti-mouse IL-2 BioLegend: https://www.biolegend.com/en-us/products/purified-anti-mouse-il-2-antibody-949
Biotinylated Anti-mouse IL-2 BioLegend: https://www.biolegend.com/en-us/products/biotin-anti-mouse-il-2-antibody-951
InVivoMab anti-mouse TIGIT BioXCell: https://bxcell.com/product/invivomab-anti-mouse-tigit/
InVivoMab anti-mouse NK1.1 BioXCell: https://bxcell.com/product/nk-1-1/
InVivoMab anti-mouse CD3 BioXCell: https://bxcell.com/product/m-cd3/
Biotinylated Anti-human IgG Jackson ImmunoResearch: https://www.jacksonimmuno.com/catalog/products/309-065-082

Eukaryotic cell lines

Policy information about cell lines

Cell line source(s)

YTS Eco Cohen et. al.(Cohen et al., 1999, PMID 10403641)
YTS TIGIT Y231A Stanietsky et. al.(Stanietsky et al., 2009, PMID 1981549)
YTS TIGIT Y231Stop Stanietsky et. al.(Stanietsky et al., 2009, PMID 1981549)
YTS TIGIT Stanietsky et. al.(Stanietsky et al., 2009, PMID 1981549)
BW5147 (Prof. Jack L. Strominger's laboratory)
BW TIGIT (Stanietsky et al., 2009, PMID 1981549)
P815 (Prof. Jack L. Strominger's laboratory)
HEK293T (Prof. Jack L. Strominger's laboratory)
RPMI-8866 (Prof. Jack L. Strominger's laboratory)

Authentication

The cell lines used were authenticated only by their manufacturer at the time of delivery and validated for the expression of their main markers by us periodically

Mycoplasma contamination

All cell lines were tested negative for mycoplasma

Commonly misidentified lines
(See ICLAC register)

No commonly misidentified lines were used in this study

Animals and other organisms

Policy information about studies involving animals; ARRIVE guidelines recommended for reporting animal research

Laboratory animals

Male C57BL/6 mice aged 6-8 weeks were used for all in-vivo experiments. The mice were test-naïve and were group-housed under specific pathogen free (SPF) conditions prior to their use. All experiments were done in the SPF unit of the Hebrew University-Hadassah Medical School (Ein-Kerem, Jerusalem) in accordance with the guidelines of the Declaration of Helsinki and the local research ethics committee. The mice were kept under constant conditions consisting of temperature of 24 celsius, 12/12 hours dark-light cycle, and 30-70% humidity.

Wild animals

The study did not involve wild animals

Field-collected samples

No field-collected samples were used in this study

Ethics oversight

All experiments were done in the SPF unit of the Hebrew University-Hadassah Medical School (Ein-Kerem, Jerusalem) in accordance with the guidelines of the Declaration of Helsinki and the local research ethics committee.

Note that full information on the approval of the study protocol must also be provided in the manuscript.

Human research participants

Policy information about studies involving human research participants

Population characteristics

The clinical Candida isolates were collected from 104 human patients suffering from bloodstream Candida infections. The population consisted of 63% men and 37% women, aged 1-94 (mean age 63).

Recruitment	Candida samples were picked randomly from a pool of clinical isolates kept within the institute. Investigators were blinded to the experimental design while choosing the isolates. As only the investigators picked the clinical samples to be analyzed, no self-selection bias was present.
Ethics oversight	The clinical trial was approved by the Tel Aviv Sourasky Medical Center Institutional Ethics Committee (approval number 0729-16).

Note that full information on the approval of the study protocol must also be provided in the manuscript.

Flow Cytometry

Plots

Confirm that:

- [x] The axis labels state the marker and fluorochrome used (e.g. CD4-FITC).
- [x] The axis scales are clearly visible. Include numbers along axes only for bottom left plot of group (a 'group' is an analysis of identical markers).
- [x] All plots are contour plots with outliers or pseudocolor plots.
- [x] A numerical value for number of cells or percentage (with statistics) is provided.

Methodology

Sample preparation	Mammalian cell lines or primary cells or fungal cells were grown as described in the manuscript's relevant methods section At the start of the experiment the cells were washed three times in 1xPBS. For each wash the conditions were 515G (for mammalian cells) or 3000G (for fungal cells), for 5 minutes in 4°C. Following the washes, the cells were counted using a hemocytometer and divided into U-bottomed 96-well plates to a concentration of 5 or 10x10^4 cells/well. Each well was incubated in the presence of primary antibodies (0.25ug/well) or Ig-fusion proteins (0.5-5ug/well) diluted in FACS medium (1x PBS, 0.05% Bovine Serum Albumin, 0.05% NaN3) for 1 hour on ice. In instances when the primary antibodies were not fluorophore-conjugated the cells were next washed one time with FACS medium, and then stained with 2nd antibodies (0.75ug/well) for 30-45 minutes on ice. Finally, the cells were washed 2 times in FACS medium, and analyzed.
Instrument	FACSCalibur machine (BD Biosciences) or a CytoFlex machine (Beckman-Coulter Life Sciences) were used for the flow cytometry experiments
Software	FCS Express software (De Novo Software) was used for the flow cytometry data analysis
Cell population abundance	No sorting was performed for this study. Most flow cytometry experiments were performed on pure cell line cultures. When investigating primary human or murine cells the purity was validated using stainings as described in the relevant methods section (CD3 and CD56 staining for human NK cells, CD4 and TIGIT staining for human T cells, and GFP expression and TIGIT staining for murine splenic NK cells).
Gating strategy	Cells were gated according to their forward and side scatter

- [x] Tick this box to confirm that a figure exemplifying the gating strategy is provided in the Supplementary Information.
